# The Mediating Role of the Muscle Quality Index in the Relation of Screen Time and Abdominal Obesity with Health-Related Quality of Life in Chilean Schoolchildren

**DOI:** 10.3390/nu15030714

**Published:** 2023-01-31

**Authors:** Pedro Delgado-Floody, Manuel Gómez-López, Felipe Caamaño-Navarrete, Pablo Valdés-Badilla, Daniel Jerez-Mayorga

**Affiliations:** 1 Department of Physical Education, Sport and Recreation, Universidad de La Frontera, Temuco 4811230, Chile; 2 Strength & Conditioning Laboratory, CTS-642 Research Group, Department Physical Education and Sports, Faculty of Sport Sciences, University of Granada, 18011 Granada, Spain; 3 Department of Physical Activity and Sport, Faculty of Sports Sciences, University of Murcia, 30720 Murcia, Spain; 4 Physical Education Career, Universidad Autónoma de Chile, Temuco 4780000, Chile; 5 Department of Physical Activity Sciences, Faculty of Education Sciences, Universidad Católica del Maule, Talca 3466706, Chile; 6 Carrera de Entrenador Deportivo, Escuela de Educación, Universidad Viña del Mar, Viña del Mar 2520000, Chile; 7 Exercise and Rehabilitation Sciences Institute, School of Physical Therapy, Faculty of Rehabilitation Sciences, Universidad Andres Bello, Santiago de Chile 7591538, Chile

**Keywords:** muscle quality index, fitness, schoolchildren, quality of life

## Abstract

Screen time (ST) and abdominal obesity have a negative effect on health-related quality of life (HRQoL). However, there is little information regarding the mediating role of the muscle quality index (MQI) in these relationships. The aim of the present study was to investigate the association between HRQoL, physical status (i.e., anthropometrics and fitness), lifestyle (i.e., ST and physical activity), and the MQI, and then to determine the potential mediating role of the MQI in the relation of ST and abdominal obesity with HRQoL in Chilean schoolchildren. The cross-sectional study included 750 schoolchildren (girls, *n* = 332 and boys, *n* = 418) aged between 10 and 14 years (11.73 ± 1.08 y). MQI, lifestyle, fitness parameters, waist-to-height ratio (WtHR) and HRQoL were measured. HRQoL presented a significant correlation with WtHR (r: −0.19), VO2_max_ (r: 0.20), physical activity after school (r: 0.26), ST (r: −0.26) and MQI (r: 0.15). According to MQI, the high-MQI group reported higher HRQoL than the low-MQI group (low MQI: 36.10 ± 3.63 vs. high MQI: 37.43 ± 4.00, *p* < 0.001). In the mediation model, ST and abdominal obesity were negatively linked to HRQoL; the indirect effect confirmed that MQI is a partial mediator in the relation between ST and HRQoL (indirect effect = −0.04; SE = 0.02; 95% CI: −0.09, −0.01) and in the relation between abdominal obesity and HRQoL (indirect effect = −1.81; SE = 0.83; 95% CI: −3.41, −0.40). In conclusion, MQI is related to better HRQoL in schoolchildren, and the negative relation of ST and abdominal obesity with HRQoL is mediated by MQI.

## 1. Introduction

Health-related quality of life (HRQoL) is commonly described as a multi-dimensional construct [1]; it is a term related to aspects of self-perceived wellbeing in the physical, mental, and social dimensions, as well as how well a person functions in their life [2]. Hence, there has been a growing concern to measure HRQoL in schoolchildren and adolescents [1], due to childhood and adolescence being critical periods of life in which many social, physiological, and psycho-emotional changes happen [3], especially in the development of self, personal, and collective identities, as well as habits that favor a positive HRQoL [4,5]. A positive wellbeing (i.e., mental health) allows an individual to develop their own abilities, to be resistant to the stresses of life, and to make a beneficial contribution to their peers [6].

In addition, HRQoL is affected by lifestyle and different anthropometrics, fatness, and fitness variables in children and adolescents. Different studies have reported that screen time (ST) affects HRQoL and mental health. In terms of ST and its relation with physical and mental health variables, evidence has suggested that higher levels of ST in children and adolescents are associated with reduced physical activity, increased risk of depression, and lower wellbeing [7,8]. A cross-sectional and longitudinal study reported that physical activity and sedentary behaviors such as television viewing, computer and video-game usage, etc., were linked to HRQL, and that subjects who spend more ST activities have a poorer HRQoL than their counterparts [9]. On the other hand, abdominal obesity has traditionally been linked to metabolic syndrome [10]; however, there is currently evidence of its impact on HRQoL dimensions [11], as students with central obesity have lower HRQoL scores in some dimensions. A study that determines the associations between the waist-to-height ratio as a measure of central obesity with health-related HRQoL in primary schoolchildren reported that lower HRQoL in children was linked to central obesity [12]. So, based on previously reported findings, the encouragement to meet ST recommendations and reduce abdominal obesity can improve HRQoL in children and adolescents [13].

Similarly, fitness has been shown to be powerful markers of health, including a greater well-being self-concept [14], and physical fitness has been linked to better HRQoL. In this sense, cardiorespiratory fitness has been positively associated with a higher score in the different domains of HRQoL; hence, it might be especially useful for improving HRQoL in children [15]. A study in Portuguese adolescents determined the relationship between cardiorespiratory fitness and muscular fitness with HRQoL, and reported that HRQoL was positively linked to cardiorespiratory fitness and muscular fitness scores controlling for potential confounders [16]. Moreover, cardiorespiratory fitness in children (6 and 8 years) moderates the relationship between the severity of life events and HRQoL, where children with higher fitness levels present higher wellbeing in physical function and report more positive relationships with friendship [17]. Moreover, evidence has shown that handgrip strength (HGS) is correlated with HRQoL dimensions and cardiovascular biomarkers [18,19], and it may be especially important when assessing markers of physical and mental health. Therefore, an evaluation of HGS and the quality of skeletal muscle is important in assessing deficient and low muscle performance, which is a key contributor to different markers of metabolic syndrome [20]. In this sense, HGS is a good measure of muscle strength and could help to recognize the risk of sarcopenic in students [21]. Likewise, in adult populations, it has been indicated that HGS is a good predictor of morbidly and mortality and could be influenced by body composition parameters [22]. In this sense, poor HGS predicts an increased risk of functional limitations in the future [23]. 

Using HGS to evaluate muscle strength has numerous advantages. In particular, compared to other tools (i.e., dual-energy X-ray absorptiometry and bioelectrical impedance), HGS can be evaluated rapidly and easily in field-based testing [20]. Likewise, the muscle quality index (MQI), which is obtained by dividing the HGS score by the body mass index (BMI), is an emerging indicator of health and physical function [18,19]. However, studies have been carried out mainly in the adult population, and there are few studies on the mediating role of the MQI in the relationship between negative health markers and lifestyle with HRQoL. Thereby, this present study aims to investigate the association between HRQoL, physical status (i.e., anthropometrics and fitness), lifestyle (i.e., ST and physical activity), and the MQI, and then to determine the potential mediating role of the MQI in relation to ST and abdominal obesity with HRQoL in Chilean schoolchildren.

## 2. Materials and Methods

### 2.1. Participants

This cross-sectional study included seven hundred and fifty students (*n* = 332 girls and *n* = 418 boys) aged between 10 and 14 years from public and subsidized schools in Temuco, Chile (Araucanía region). Guardians of the participants were asked to give signed consent and assent of the children before involvement in this investigation. The sample was intentional and non-probabilistic.

The inclusion criteria were: (i) assent of the children and informed consent of the guardians; (ii) the participant belonged to the school; and (iii) the participant was aged between 10–14 years. The exclusion criteria were: (i) presentation of a musculoskeletal disorder; or (ii) having any other known condition (medical). Likewise, participants with physical, sensory, or intellectual disabilities were excluded from this investigation. This study complied with the Helsinki Declaration (2013) and was approved by the Ethics Committee of Universidad de La Frontera, Chile (ACTAN 086_2017). This investigation corresponds to a part of a research project (DFP16-0013; 2016–2017, DIUFRO, TEMUCO, CHILE). The researchers´ team who evaluated the students were trained in protocols of different tests and evaluations. In the first session, the anthropometric and health variables were carried out in a comfortable room facilitated by the educational centers, with good temperature and privacy. The questionaries and surveys, such as KIDSCREEN and the Krece Plus test, were applied on different days to the anthropometric evaluations in classrooms. The questionnaires were completed individually and in the presence of researchers. In the last session, the physical fitness evaluations took place during physical education classes, one for cardiorespiratory fitness and the other for muscular strength.

### 2.2. Main Outcomes

#### 2.2.1. Health-Related Quality of Life

KIDSCREEN-10 was used to evaluate HRQoL. The KIDSCREEN-10 is the brief form of a measure originally developed in Europe, for aged 8–18 years (children and adolescents). The KIDSCREEN questionary has been validated in many countries [24], and it has been validated in Chilean children and adolescents [25,26]. It has been used to determine the relation between children’s lifestyles (i.e., physical activity, ST and sedentary behaviors), anthropometrics’ parameters and HRQoL [27,28]. KIDSCREEN-10 has 10 items, each answered on a five-point Likert scale indicating the frequency of a specific behaviour or feeling (1 = never; 2 = almost never; 3 = sometimes; 4 = almost always; and 5 = always) or the intensity of an attitude (1 = not at all; 2 = slightly; 3 = moderately; 4 = very; and 5 = extremely). The responses to items negatively formulated (i.e., items 3 and 4) were recoded to have scores from 1 to 5; the raw scores were used to analyse differences. Higher values indicate a higher HRQoL [29].

#### 2.2.2. Muscle Quality Index

HGS was evaluated by a hand dynamometer (TKK 5101^TM^, Grip D; Takei, Tokyo, Japan) in order to determine upper-body strength. The protocol, based on previous recommendations, consists of holding a dynamometer in one hand and squeezing as tightly as possible without allowing the dynamometer to touch the body and the participants occupied the same dynamometer [30]. Force was applied progressively for 3–5 s [31]. Each students performed three attempts for each hand, with a rest of 120 s between each attempt. A blinded evaluator recorded the three values, and the maximum one was used. The mean of the scores performed by the left and right hands was recorded and used in the analysis. MQI was calculated by dividing HGS by BMI, and we defined low MQI as ≤50th percentile and high MQI as >50th percentile (1.078 ratio).

#### 2.2.3. Abdominal Obesity

Waist circumference (WC) was evaluated employing a Seca^®^ tape measure model 201 (Hamburg, Germany) in agreement with standardized protocols [32]. Waist-to-height ratio (WtHR) was determined according to the established formula (WC/height) and was employed as a tool for establishing the abdominal obesity using universal standards [33]. Continuing with the above in a complementary way, a cut-off of ≥0.54 is ideal for considering cardiometabolic risk for the Latin American locality in children.

#### 2.2.4. Screen Time and Physical Activity

The participant’s lifestyle was obtained by the Krece Plus test [34]. This instrument is a questionnaire that classifies lifestyle dimensions according to the average amount of ST daily (i.e., watching television or playing video games) and PA after school per week. The categorization is determined according to the number of hours for each item. The total score is added up, and the subject is classified according to the following dimensions: good lifestyle (male ≥ 9 h, female ≥ 8 h), regular lifestyle (male 6–8 h, female 5–7 h) or bad lifestyle (male ≤ 5 h, female ≤ 4 h). This instrument has previously been used in Chilean children and adolescents [35].

#### 2.2.5. Cardiorespiratory Fitness

Cardiorespiratory fitness was evaluated by the 20 m shuttle run test (20mSRT) following international indications [36]. The test has been performed in the National Physical Education study and validated in Chilean schoolchildren [37]. The outcomes obtained from the 20mSRT were unified according to the Leger test protocol, and the VO_2max_ was determined using Leger’s equation [36]: VO_2max_ = (31.025 + 3.238 (V) − 3.248 (A) + 0.1536 (VA)), where V is the velocity in km/h reached in the last stage, and A represents the age of the subject [38]. Good values are equal to or higher than 42 mL/kg/min, whereas low values are less than 42 mL/kg/min, according to age and sex [38].

#### 2.2.6. Anthropometric Parameters

The participants’ body mass (kg) was determined using the TANITA model Scale Plus UM–028 scales (Tokyo, Japan). The height (cm) was evaluated with a Seca^®^ model 214 stadiometer (Hamburg, Germany), graded in mm. body mass index (BMI) was estimated as body mass (kg) divided by the square of the height in metres (kg/m^2^) [39].

### 2.3. Statistical Analysis

Normal distribution was conducted using the Kolmogorov–Smirnov. Descriptive data was presented in terms of means and standard deviation (SD). Differences between mean values according to sex and MQI (in HRQoL) were established using ANOVA and the chi-square test, respectively. The relationship between lifestyle, physical variables and HRQoL was calculated with Pearson’s correlation coefficient (r). Regression analyses were performed to verify the effect of the variables mediating MQI (M), considering ST and abdominal obesity (WtHR) as independent variables (X) and HRQoL as a dependent variable (Y); sex and age were included as covariables in the model (model 2) to determine any changes in the associations. Within the analysis, the total effect (c), direct effect (c′) and indirect effect (a * b, IE) were calculated for the samples as well as the 95% confidence interval (CI) using the macro/interface process v. 3.3 for SPSS v. 23 and the bootstrapping method with a resampling rate of 5000 [40]. All the statistical analyses were performed with SPSS statistical software version 23.0 (SPSS^TM^ Inc., Chicago, IL, USA). The alpha level was set at *p* < 0.05 for statistical significance.

## 3. Results

Table 1 shows a comparison of the study variables according to sex. There were significant differences in VO_2MAX_ (*p* < 0.001), HGS (*p* = 0.001) and MQI (*p* < 0.001) (Table 1).

In the correlation analyses, HRQoL presented a significant relation with BMI (r: −0.14), WtHR (r: −0.19), VO_2max_ (r: 0.20), physical activity after school (r: 0.26), ST (r: −0.25) and MQI (r: 0.14) (Table 2).

**Figure 1:**
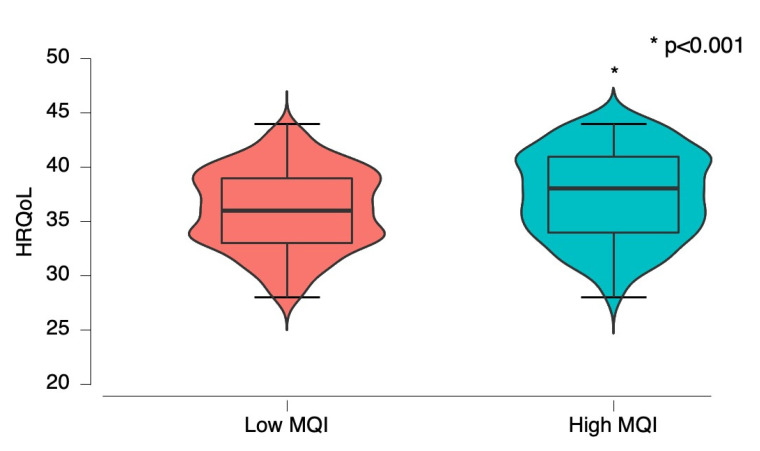
Health related to quality of life (HRQoL) according to muscle quality index (MQI); low MQI vs. high MQI.

According to MQI levels, high-MQI groups reported higher HRQoL than the low-MQI group (low MQI: 36.10 ± 3.63 vs. high MQI: 37.43 ± 4.00, *p* < 0.001) (Figure 1).

**Figure 2:**
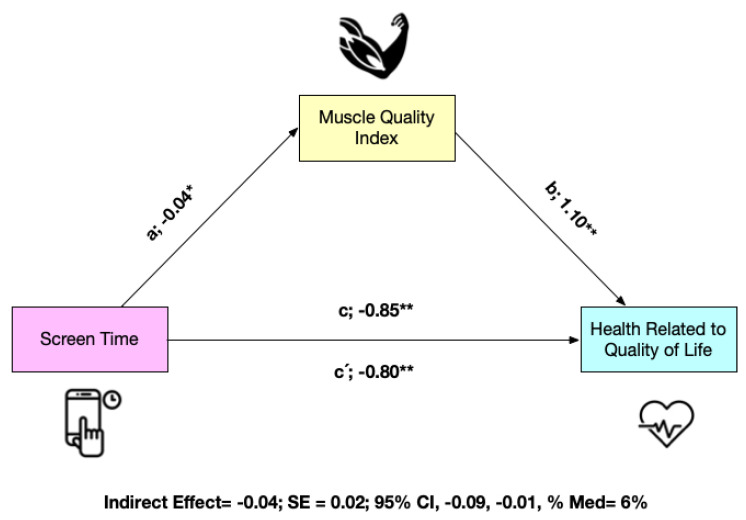
Mediation model testing whether the association between screen time and health related to quality of life was mediated by muscle quality index * *p* < 0.05; ** *p* < 0.001. Panel A non-adjusted, Panel B adjusted by sex and age.

The mediation analysis is shown in Figure 2 for the total sample (*n* = 750 schoolchildren). MQI appears as a mediating variable in the relationship between ST and HRQoL. In the first regression step (a), ST was inversely related to MQI (*p* < 0.05). In the second step (c), the regression coefficient of ST in HRQoL was also significant (*p* < 0.001). In the third step, the potential mediator MQI was positively related to the dependent variable (b) (*p* < 0.05), but when both ST and MQI were included in the model (c′), the regression coefficient remained statistically significant (*p* < 0.001). Finally, the indirect effect confirms that MQI is a partial mediator of HRQoL (indirect effect = −0.04; SE = 0.02; 95% CI: −0.09, −0.01) (Figure 2). 

**Figure 3:**
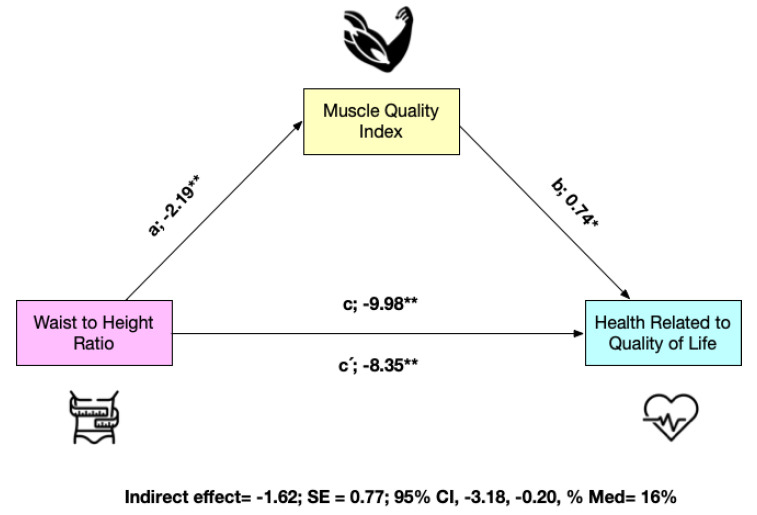
Mediation model testing whether the association between waist-to-height ratio and health related to quality of life was mediated by muscle quality index. * *p* < 0.05; ** *p* < 0.001. Panel A non-adjusted, Panel B adjusted by sex and age.

Considering abdominal obesity (WtHR), in the first regression step (a), it was inversely related to MQI (*p* < 0.001). In the second step (c), the regression coefficient of abdominal obesity in HRQoL was also significant (*p* < 0.001). In the third step, the potential mediator MQI was positively related to the dependent variable HRQoL (b) (*p* < 0.001). Finally, the indirect effect confirms that MQI is a partial mediator of HRQoL (indirect effect = −1.62; SE = 0.77; 95% CI: −3.18, −0.20) (Figure 3). When the model was adjusted for sex and age, the results remained the same.

## 4. Discussion

In the present study, the aim was to investigate the association between HRQoL, physical status (i.e., anthropometrics and fitness), lifestyle (i.e., ST and PA), and the MQI, and then to determine the potential mediating role of the MQI in the relation of ST and abdominal obesity with HRQoL in Chilean schoolchildren. The main results of the present study were as follows: (i) there was an association between ST and abdominal obesity with HRQoL in children; (ii) the MQI had a mediating role in these relationships; and (iii) cardiorespiratory fitness was related to HRQoL. 

The MQI played a mediating role in the relation between ST and HRQoL in Chilean schoolchildren: the correlation analyses showed that HRQoL is inversely related to ST. Consistently, ST has been negatively linked with mental health and HRQoL [7,41]. Students who exceeded 2 h of ST a day had lower HRQoL scores [42]. Despite the evidence linking ST with poorer health, schoolchildren and adolescents do not meet ST recommendations [43]. For example, it has been reported in Chilean adolescents that an unhealthy lifestyle that included high ST was related to low HRQoL. Moreover, PA after school and Mediterranean diet adherence positively mediated the link between self-esteem and HRQoL; on the contrary, ST negatively mediated this relation [27]. In this sense, a previous study showed that high ST and bad food habits were related with poorer mental health; likewise, this study reported that physical fitness dimensions were positively linked with the absence of body image dissatisfaction [14]. Likewise, a cross-sectional study conducted in children indicated that excessive ST was linked with a high risk of internalizing and externalizing mental health problems; therefore, the authors concluded that it is fundamental to develop healthy lifestyle interventions [44]. In addition, a systematic review which examined the longitudinal relation between ST and mental health reported that recreational ST was negatively linked with psychological wellbeing [45]. On the contrary, the evidence highlights the positive role of muscle fitness in HRQoL in school-aged children [46]. In this sense, a study conducted in adolescents reported that physical fitness that included muscular strength and cardiorespiratory fitness was positively linked with HRQoL; therefore, physical fitness components could be an important factor in improving subjective wellbeing [47]. Another study showed that subjects with high physical fitness (i.e., HGS, cardiorespiratory fitness and motor fitness) and high Mediterranean diet adherence (i.e., KIDMED index) had better HRQoL compared with their counterparts [46]. In addition, it has been reported that strength in the lower body was positively linked with some dimensions of HRQoL (i.e., higher autonomy and parents score) in children [15]. Moreover, data from Spanish children and adolescents showed that participants who had better HRQoL obtained optimal levels of muscle strength [48]. In addition, another study conducted in Portuguese adolescents showed that muscular fitness scores (i.e., the mean HGS score and the standing long jump test results) were positively related with HRQoL. In addition, students with high muscular fitness and cardiorespiratory fitness had better HRQoL than those with poor physical fitness; therefore, fitness levels could be a good indicator for HRQoL [16]. 

Continuing with the above, in the present study, cardiorespiratory fitness was related to HRQoL in the sample study. In this sense, a previous investigation reported that physical fitness mediates the association between physical activity and HRQoL, muscular fitness, and cardiorespiratory fitness by predicting the mental wellbeing dimensions of HRQoL in schoolchildren [49]. Likewise, it has been indicated that healthy lifestyle was positively related with mental health (i.e., self-esteem) and HRQoL [27]. Likewise, it has been shown that bad cardiorespiratory fitness was linked with poor physical self-concept in Chilean schoolchildren [14]. Another study reported that cardiorespiratory fitness was linked with mental health, therefore promoting physical fitness may improve adolescent’s wellbeing [50]. Moreover, it has been indicated that a high fitness status, which includes muscular strength, is linked with improved HRQoL in Spanish students [48]. Additionally, it has been demonstrated that muscular fitness and cardiorespiratory fitness mediate the association between a healthy lifestyle (i.e., ST, physical activity, and Mediterranean diet adherence) and body dissatisfaction; therefore, adolescents with better physical fitness may have fewer mental health problems [14,51] and better HRQoL [52]. Another study showed a positive relation between cardiorespiratory and muscular fitness with mental health and HRQoL [53]. In this sense, a previous study conducted in Serbian adolescents reported that high cardiorespiratory fitness was related with a better HRQoL score and psychological wellbeing [54].

On the other hand, in this study, abdominal obesity (i.e., WtHR) was inversely related to HRQoL, and the MQI of schoolchildren played a mediating role in this relation. In this sense, another study reported poorer HRQoL in schoolchildren who had more central obesity classified by WtHR [12]. Similarly, a cross-sectional study that evaluated the associations between central obesity and HRQoL showed that central obesity is linked to different HRQoL dimensions [55]. In this way, there is evidence that the negative link between weight status and poor HRQoL is mediated by physical fitness that includes HGS, countermovement jumps, core body strength, and agility [56]. Likewise, a systematic review showed a significant reduction in HRQoL in obese youths [57]. Moreover, in data from seven Italian schools, obesity categories were negatively linked with HRQoL dimensions, with the relations being more pronounced in females than in males [58]. Furthermore, a study conducted in overweight and obese schoolchildren reported that improvements in HRQoL after the application of physical activity intervention were mediated by a reduction in central obesity [13]. On the other hand, a negative association between physical fitness and central obesity has been reported [59]. Another study that measured the relationship between adiposity and muscular strength in Chilean students showed that participants with a risk of abdominal obesity had increased risk of poor relative HGS (i.e., the MQI) [60]. Likewise, another study showed that HGS is related to lower body fat in schoolchildren [61]. Improvements in HGS could also promote better HRQoL.

In the present study, children with a high MQI reported a higher score in HRQoL than their counterparts. In this sense, another study conducted in schoolchildren reported that explosive strength is positively linked with some dimensions of HRQoL, such as higher autonomy and parents’ score [15]. In addition, it has been indicated that poor HGS is linked with discomfort dimensions of HRQoL [62]. Another example of this is a study that showed that HGS is related to the social support and peer dimensions of HRQoL [63]; therefore, it is fundamental to consider physical fitness as a means to improve the dimensions of HRQoL. In addition, another study reported that physical fitness is more linked with self-perception and social confidence [56].

Possible limitations of the study include (i) not including assessments of neurophysiological mechanisms to determine the activation of flexor–extensor muscles implicated in the evaluation of physical fitness, (ii) as well as the selection of the sample (non-probabilistic). Likewise, another limitation is the cross-sectional design. Among the strengths, we could indicate: (i) utilize the questionnaires and protocols that have been validated in the Chilean context to assess schoolchildren or (ii) the simplicity of the assessments (which would allow their use and implementation in healthy lifestyle programs focused on children and adolescents). 

## 5. Conclusions

In conclusion, the MQI is associated with better HRQoL in schoolchildren, and the negative relation between ST and abdominal obesity with HRQoL is mediated by the MQI. Thus, the MQI is an important measure to consider as a variable of not only physical health but also wellbeing in schoolchildren. Moreover, cardiorespiratory fitness was positively related to HRQoL; therefore, promoting a positive physical fitness and weight status among students should be a target of community- and school-based interventions in order to promote a better wellbeing. These findings provide evidence that improving the MQI and cardiorespiratory fitness might be a practical strategy to improve HRQoL in children. Thus, the educational community should incorporate effective strategies and interventions that generate opportunities inside and outside school that aim to jointly improve general wellbeing.

## Figures and Tables

**Table 1:** Characteristics of sample study according to sex.

	Total (*n* = 750)	Girls (*n* = 332)	Boys (*n* = 418)	*p* Value _(F-value)_
Age (years)	11.65 ± 1.13	11.62 ± 1.08	11.66 ± 1.16	0.637 _(0.22)_
Height (cm)	155 ± 0.11	154 ± 0.09	156 ± 0.11	0.090 _(7.13)_
Body mass (kg)	53.23 ± 14.40	52.82 ± 13.49	53.55 ± 15.09	0.494_(0.47)_
BMI (kg/m^2^)	21.89 ± 4.63	22.10 ± 4.68	21.73 ± 4.59	0.273 _(1.20)_
WC (cm)	74.21 ± 12.08	73.32 ± 11.44	74.92 ± 12.53	0.093 _(2.83)_
WtHR (WC/size)	0.48 ± 0.07	0.48 ± 0.07	0.48 ± 0.07	0.443 _(0.59)_
Abdominal obesity				
No	602 (80.3%)	274 (82.5%)	328 (78.5%)	*p* = 0.097
Yes	148 (19.7%)	58 (17.5%)	90 (21.5%)	
VO2_max_ (ml/kg/min)	44.34 ± 5.57	42.77 ± 4.63	45.57 ± 5.93	*p* < 0.001 _(49.94)_
HGS (kg)	23.84 ± 7.94	22.76 ± 7.91	24.71 ± 7.88	*p* = 0.001 _(11.33)_
MQI (HGS/BMI)	1.14 ± 0.44	1.07 ± 0.42	1.19 ± 0.45	*p* < 0.001 _(13.00)_
Low MQI	373 (49.7%)	184 (55.4%)	189 (45.2%)	*p* = 0.003
High MQI	377 (50.3%)	148 (44.6%)	229 (54.8%)	
Screen time (h/day)	3.39 ± 1.12	3.45 ± 1.14	3.34 ± 1.11	0.221 _(1.50)_
PA after school (h/week)	2.45 ± 1.38	2.36 ± 1.32	2.51 ± 1.42	0.139 _(2.19)_
HRQoL (score)	36.77 ± 3.87	36.76 ± 3.83	36.78 ± 3.91	0.940 _(0.01)_

Data are presented as mean and standard deviations (SD). *p* < 0.05 considered statistically significant. BMI = body max index, WC = waist circumference, WtHR = waist-to-height ratio, VO2_max_ = maximal oxygen consumption, ST = screen time, PA = physical activity, MQI = muscle quality index, HRQoL = health-related quality of life.

**Table 2:** Correlation among anthropometrics, abdominal obesity, fitness and health-related quality of life.

	BMI (kg/m^2^)	WtHR (WC/size)	VO2_max_ (ml/kg/min)	Screen Time (h/day)	PA after School (h/week)	MQI (Ratio)	HRQoL (Score)
BMI (kg/m^2^)	-						
WtHR (WC/size)	0.70 **						
VO2_max_ (ml/kg/min)	−0.33 **	−0.26 **					
Screen time (h/day)	0.20 **	0.15 **	−0.14 **				
PA after school (h/week)	−0.26 **	−0.25 **	0.29 **	−0.63 **			
MQI (ratio)	−0.50 **	−0.30 **	0.31 **	−0.10 **	0.17 **		
HRQoL (score)	−0.14 **	−0.19 **	0.20 **	−0.25 **	0.26 **	0.14 **	-

Data are presented as Pearson coefficient correlation (r). *p* < 0.05 considered statistically significant, ** represents *p* < 0.01. BMI = body max index, WtHR = waist-to-height ratio, VO2_max_ = maximal oxygen consumption, ST = screen time, PA = physical activity, MQI = muscle quality index, HRQoL = health-related quality of life.

## Data Availability

Not applicable.
